# Examining the promotive versus the protective impact of culturally informed shift‐&‐persist coping in the context of discrimination, anxiety, and health behaviors

**DOI:** 10.1002/jcop.22799

**Published:** 2022-01-20

**Authors:** N. Keita Christophe, Michelle Y. Martin Romero, Gabriela L. Stein

**Affiliations:** ^1^ Department of Psychology Wake Forest University Winston‐Salem North Carolina USA; ^2^ Department of Public Health Education University of North Carolina at Greensboro Greensboro North Carolina USA; ^3^ Department of Psychology The University of North Carolina at Greensboro Greensboro North Carolina USA

**Keywords:** anxiety, coping, discrimination, shift‐&‐persist, sleep

## Abstract

This study aims to better understand how racially/ethnically minoritized youth exhibit adaptive psychological functioning (less anxiety) and health behaviors (better sleep and less binge drinking) in the context of discrimination, ethnic‐racial identity and coping. Among 364 minoritized emerging adults (*M*_age_ = 18.79, 85.2% female), we utilized higher‐order factor analysis to examine how culturally informed shift‐&‐persist (S&P), a higher‐order construct explaining associations between coping factors (shift, persist, spiritually based coping, civic engagement), and ethnic‐racial identity were related to anxiety, binge drinking, and sleep in the context of discrimination. Culturally informed S&P promoted better sleep and less anxiety controlling for discrimination. No significant effects were observed for binge drinking and no moderated effects were observed across outcomes. The harmful effect of discrimination on sleep was intensified for those with stronger ethnic‐racial identities. The promotive and potentially protective effects of culturally informed S&P coping differs across mental health and health behavior outcomes.

## 1 INTRODUCTION

Discrimination is largely an uncontrollable stressor that exerts a harmful and wide‐reaching influence on the psychological health, physical health, and health behaviors of racially/ethnically minoritized^1^ individuals (Priest et al., 2013). Previous theoretical work suggests that the coping strategies minoritized individuals employ to combat discrimination may be more effective if they are aligned with the individual's cultural beliefs, ethnic‐racial identity (ERI), and context (Heppner et al., 2014). While some scholarship has worked to empirically test these theoretical claims, quantitative scholarship on the promotive (i.e., associated with a desirable outcome as the main effect) and protective (i.e., reducing the harmful impact of a stressor on an outcome; Masten et al., 2009) nature of culturally informed coping in the context of discrimination is both lacking and largely restricted to the study of depressive symptomatology. Discrimination may, indeed, impact other psychological outcomes, such as anxiety (Priest et al., 2013), as well as impact health behaviors such as sleep (Slopen et al., 2016) and alcohol use (Gilbert & Zemore, 2016). This study utilized a sample of minoritized college students to examine how endorsement of a strong ERI and individual's levels of culturally informed shift‐&‐persist (S&P) coping are related to anxiety symptoms and two important health behaviors: sleep and binge drinking.

## 2 DISCRIMINATION AND ANXIETY

Discrimination is a persistent predictor of greater anxiety symptoms among marginalized individuals (Priest et al., 2013), and feelings of anxiety are often an immediate psychological stress response for those exposed to discrimination (Clark et al., 1999). Despite the robust and long‐lasting impact of discrimination on anxiety symptoms, studies of the effects of discrimination on mental health tend to focus less on anxiety (Priest et al., 2013), and studies of cultural promotive and protective factors in the context of discrimination are rarer still.

ERI and culturally informed coping processes (Heppner et al., 2014) may play a role in promoting positive development among minoritized youth. For instance, in a meta‐analysis of the effects of religious coping, a form of coping often rooted in the cultural values and beliefs of minoritized groups (Knight et al., 2010), Ano and Vasconcelles (2005) found a negative association between positive religious coping and anxiety symptoms. By contrast, in their review on sociocultural factors influencing anxiety in African Americans, Hopkins and Shook (2017) found mixed evidence for the promotive impact of spiritually based/religious coping and consistent evidence for the promotive impacts of ERI and the harmful impacts of discrimination. However, this review does not address whether and how cultural factors such as ERI and culturally informed ways of coping may interact with each other to predict anxiety, nor does it distinguish whether the promotive effects of coping or ERI hold when considering the impact of discrimination.

### 2.1 Discrimination and sleep

A burgeoning body of literature has demonstrated a link between discrimination and negative sleep outcomes (e.g., shorter sleep duration, poorer sleep quality) among minoritized individuals across multiple developmental stages (Zeiders et al., 2017). In a systematic review of the relationship between discrimination and sleep, discrimination was associated with poorer sleep across all 17 studies examined (Slopen et al., 2016). Given that sleep is a biological need that is critical for optimal development and adjustment across the life course, it is necessary to identify potential factors that might mitigate the impact of discrimination on sleep and/or promote greater sleep health in the face of discrimination. Preliminary work suggests that ERI and culturally informed coping may serve these needed promotive and protective functions. For example, Cheon et al. (2019) found ERI commitment to be directly associated with sleep duration and quality among a multiethnic sample of adolescents. Further, in a sample of 246 Mexican‐origin young adults (*M*_age_ = 21.11 years), bicultural (oriented toward one's heritage cultural group and mainstream Eurocentric culture), and enculturated (oriented solely toward one's heritage group) orientations moderated the relationship between discrimination and sleep duration (Zeiders et al., 2017). Conversely, for young adults in this sample who were more acculturated (Anglo mainstream orientation), exposure to discrimination negatively impacted sleep duration. Although limited, these preliminary findings underscore the need to include cultural factors in the study of discrimination and health behaviors like sleep, with the hope of informing future interventions.

### 2.2 Discrimination and alcohol use

Theory asserts that substance use serves to dampen the stress associated with discrimination (Neblett et al., 2010). This assertation is supported by recent work by Metzger et al. (2018), whereby the perceived stressfulness of discrimination moderated the link between discrimination and alcohol use in Black college students such that those who reported more stress associated with discrimination also reported the most alcohol use. Discrimination has also been documented as a risk for greater substance use in larger multi‐ethnic samples and with Latinx participants in longitudinal studies across adolescence and emerging adulthood (e.g., Unger et al., 2014). ERI processes may serve as a buffer of this harmful link between discrimination and substance use (Gibbons & Stock, 2018). Coping that is embedded with a racial self‐concept and strengthens how youth deal with racialized stressors may, in addition to ERI, also be particularly beneficial (Neblett et al., 2010). Although neither private regard nor centrality mitigated the harmful effects of discrimination on substance use in a Black adolescent sample (Fuller‐Rowell et al., 2012), ERI has been identified as a promotive factor predicting less overall substance use across racial/ethnic groups (Pugh & Bry, 2007). Further, work in this area has typically focused on substance use coping (e.g., Gerrard et al., 2012) and has not tested whether other ways of coping, such as culturally informed coping, may act as a buffer against the effects of discrimination on substance use in emerging adulthood.

### 2.3 Conceptual framework to understand resilience in the face of discrimination

While it is important to document and understand the persistent and harmful effects discrimination may have you minoritized youth's functioning (e.g., Benner et al., 2018), it is even more important to understand ways in which youth may show resilience in the face of discrimination. The present study is thus guided by Neblett et al.'s (2012) conceptual model, a testable model that theorized complex relations between factors that may foster positive adaptation in minoritized youth and resilience in the face of discrimination. In this model, Neblett et al. (2012) theorize interrelations between and among cultural factors, such as ERI, as well as more “normative”’ factors, such as coping; these factors are proposed to work in tandem to promote better functioning and to mitigate discrimination's negative impact on functioning. This conceptual model makes clear the complex relations between cultural and more normative factors and underscores the importance of assessing multiple factors at once to gain a more holistic understanding of how minoritized youth demonstrate resilience in the face of discrimination. In the present study, we use second‐order confirmatory factor analysis and regression models to test the joint impact of several factors outlined in Neblett et al. (2012)—specifically three related coping mechanisms (S&P, spiritually based coping, and civic engagement) and ERI—and their ability to promote positive functioning and protect against the harmful effects of discrimination. Although this study does not test the entirety of Neblett et al.'s conceptual model and the complete list of potential relevant resilience factors, it does: provide a test of the theorized associations between several cultural and normative resilience factors, tests whether culturally informed S&P—the underlying factor linking together those resilience factors—promotes adaptive outcomes, and examines whether culturally informed S&P operates differently on different youth psychosocial outcomes and health behaviors impacted by discrimination.

### 2.4 Culturally informed S&P paradigm of coping

S&P is a coping strategy proposed to protect against negative physical health outcomes (Chen et al., 2015), maladaptive health behaviors (Mello et al., 2019), and depressive symptoms (Christophe et al., 2019) for individuals exposed to uncontrollable SES and discrimination‐related stressors. S&P involves two interrelated coping actions, where the use of both in tandem has been theorized and empirically shown (Chen, Miller, et al., 2012) to be key in the effectiveness of this coping strategy. When faced with an uncontrollable stressor, youth *shift* by employing cognitive reappraisal and acceptance of the uncontrollable nature of the stressor then *persist* by maintaining a sense of purpose in life and optimism (Chen & Miller, 2012). Ways of coping that are informed by the cultural identities and beliefs of marginalized populations may embody some of the core components of S&P by helping marginalized youth shift away from stress and persist by findings meaning and optimism. Civic engagement (Hope & Spencer, 2017) and religious coping (Knight et al., 2010) are two examples of culturally informed coping with racialized stress that stem from the cultural traditions of minoritized groups and may represent shifting and persisting against discrimination. The act of civic engagement has been shown to be a driver of meaning and purpose for those from marginalized groups (Sumner et al., 2018). Similarly, spiritually based coping may also be enacted in a way consistent with S&P with marginalized individuals reappraising and accepting instances of discrimination as part of the plan of a higher power and subsequently finding purpose, optimism, and hope for the future in their relationship with their faith/spiritual community (Christophe et al., 2021).

In the sample of 364 minoritized emerging adults examined in the current study, associations between shift, persist, civic engagement, and spiritually based coping were explained through a higher‐order coping factor representing the commonalities among all these ways of coping (Christophe et al., 2021). This factor, coined culturally informed S&P, represents the unobserved way of coping that embodies cognitive reappraisal, acceptance, and purpose in life with the potentially promotive and protective aspects of youth's cultural identities that are expressed through coping. In this sample, greater culturally informed S&P coping was associated with fewer depressive symptoms and, for youth who engaged in high levels of this style of coping, discrimination was no longer a risk factor for depressive symptoms (Christophe et al., 2021). Finally, we found that ERI was no longer negatively associated with depression when controlling for culturally informed S&P, lending credence to the claim that promotive aspects of ERI on depression may be accounted for by assessing the culturally informed S&P construct.

### 2.5 Current study

Building upon our previous work, the current study sought to examine how discrimination, culturally informed S&P coping, ERI, and their two‐ and three‐way interactions predicted anxiety symptoms and two health behaviors: binge drinking and quality sleep. We hypothesized that, among our sample of minoritized college students, culturally informed S&P will moderate the associations between discrimination and two of our outcomes, binge drinking and quality sleep, such that discrimination will no longer be associated with greater binge drinking and less quality sleep when participants are high in culturally informed S&P coping. We hypothesized that culturally informed S&P would not moderate the association between discrimination and anxiety symptoms. Across our three regression models (one for each outcome), we hypothesized that we would observe these effects when controlling for ERI. We also did not hypothesize the existence of any other significant two‐ or three‐way interactions predicting any of our outcomes (see preregistration plan; https://osf.io/5vmqc/?view_only=6e65bf878ade4bb5b418de192ae56ade).

## 3 METHOD

### 3.1 Participants and procedure

Participants included a sample of 364 racially/ethnically minoritized college students (*M*_age_ = 18.79, SD = 1.34, range = 18–26, 85.2% female, 84.7% US‐born) recruited for Christophe et al. (2021) from a public, Minority Serving Institution in the southeastern United States. Most participants self‐identified as Black (54.4%) and Latinx (13.5%), with smaller numbers of Multiracial (13.5%), Asian (8.2%), and Native American/Middle Eastern or North African/other participants (1.6%). Average social class, assessed on a 1–10 scale using the MacArthur Subjective Social Status Ladder Community Version (Adler et al., 2000) was 5.42 (SD = 1.93). After receiving institutional review board (IRB) approval, participants completed an online Qualtrics survey assessing psychological functioning, coping, ERI, and experiences of discrimination. Participants received research credits for their participation and became eligible for entry into a follow‐up study.

### 3.2 Measures

#### 3.2.1 S&P

Past frequency of shift (five items) and persist (seven items) coping were separately assessed using Lam et al.'s (2018) S&P measure. On a 1 (*Not at all*) to 4 (*A lot*) scale, participants indicated the degree to which they generally identified with items such as “When something stressful happens in my life, I think about the positive aspects or the good that could come from the situation” for shift^2^ and “I feel my life has a sense of purpose” for persist. Higher values indicated more frequent shifting and more frequent persisting. In a diverse sample of youth, reliabilities were 0.81 for shift and 0.72 for persist (Lam et al., 2018). In our sample, reliabilities were 0.75 and 0.85 for shift and persist, respectively.

#### 3.2.2 Civic engagement

Civic engagement over the past year was assessed using the four‐item communal action subscale of the Antiracism Action Scale (Aldana et al., 2019). On a binary scale, participants indicated whether they had done things such as “attended a meeting on an issue related to race, ethnicity, discrimination, and/or segregation” over the past year. Reliability in a diverse sample of adolescents was 0.65 (Aldana et al., 2019) and, as previously reported in (Christophe et al., 2021), reliability in our diverse sample of minoritized college students was 0.69.

#### 3.2.3 Spiritually based coping

Past frequency of spiritually based coping was assessed using the four‐item Relationship with God subscale of an adapted youth version of the Africultural Coping Systems Inventory (Gaylord‐Harden & Utsey, 2007). In this study, items such as “I pray or talk to God” and “I ask God for strength” were introduced with the item stem “When I face racism, discrimination, or unfair treatment.” Items were rated on a 1 (*Not at all*) to 4 (*A lot*) scale, with greater numbers indicating more spiritually based coping. When examined in combination with the three‐item “spiritual activities subscale,” reliability was 0.85 among a sample of Black adolescents (Gaylord‐Harden & Cunningham, 2009). Reliability in our sample was 0.88.

#### 3.2.4 Discrimination

Past frequency of exposure to discrimination was assessed using a mean score of the nine‐item Everyday Discrimination Scale (Williams et al., 1997). On a 1 (*Never*) to 6 *(Almost everyday*) scale, participants indicated how frequently they experienced events such as “people act as if they are afraid of you” and “you are treated with less courtesy than other people are.” Previous reliability in samples of Black and Latinx adults were 0.86 and 0.79, respectively (Krieger et al., 2005). Reliability in our sample of minoritized college students was 0.90.

#### 3.2.5 ERI

Stable, general levels of ERI endorsement were assessed by averaging scores on the four‐item centrality and four‐item private regard subscales from a version of the Multidimensional Inventory of Black Identity (MIBI; Sellers et al., 1998) adapted for use across ethnic/racial groups. On a 1 (*Strongly disagree*) to 5 (*Strongly agree*) scale, participants indicated their agreement with sample items such as “In general, being a member of my ethnic‐racial group is an important part of my self‐image" (centrality) and “I have a lot of pride in my ethnic‐racial group and its accomplishments” (private regard). Higher values indicate a stronger ERI. As previously reported, the reliability of this eight‐item ERI composite was 0.94 in our sample of minoritized emerging adults.

#### 3.2.6 Anxiety symptoms

Anxiety symptoms over the past 7 days were assessed using the seven‐item anxiety subscale from the 21‐item Depression, Anxiety, and Stress Scale (Lovibond & Lovibond, 1995). On a 0 (*Did not apply to me at all*) to 3 (*Applied to me very much or most of the time*) scale, participants indicate the degree to which items such as “I felt scared without any good reason” and “I was worried about situations in which I might panic and make a fool of myself” applied to them over the past week. Scores were summed and multiplied by two, consistent with scoring procedures (Lovibond & Lovibond, 1995), such that higher scores indicated greater anxiety. Past reliability in a diverse sample of college students (Osman et al., 2012) and reliability in our sample were both 0.81.

#### 3.2.7 Quality sleep

Quality sleep over the past 7 days was assessed using a single item, “in the past 7 days, how often have you gotten enough sleep so that you felt rested when you woke up in the morning?,” from the annual National College Health Assessment (American College Health Association, 2009). Consistent with the National College Health Assessment, categorical response options ranged from “1 day” (1) to “7 days” (1) and did not include a “0 days” response option. Greater values on this categorical item indicated more frequent quality sleep.

#### 3.2.8 Binge drinking

Binge drinking frequency over the past 2 weeks was assessed using a single‐item measure from the Monitoring the Future study (Johnston et al., 2017). Participants were told “think back over the last 2 weeks” and asked, “How many times have you had five or more drinks in a row?” with a drink being defined as “a bottle of beer, a glass of wine, a wine cooler, a shot of liquor, or a mixed drink.” Participants indicated the frequency of their binge drinking on a categorical 1–6 scale. Response options in ascending order were *None* (1), *Once* (2), *Twice* (3), 3–5 *times* (4), 6–9 *times* (5), and 10+ *times* (6).

## 4 RESULTS

### 4.1 Analytic plan

The process by which we created our higher‐order factor model using this same sample and evaluated it against similar nested factor models has been described extensively in Christophe et al. (2021). To summarize, we first evaluated the fit of each factor (shift, persist, civic engagement, and spiritually based coping) individually. Three reverse‐coded items from the persist subscale were allowed to correlate. Next, the fit of the higher‐order model was evaluated via *χ* ^2^ difference test relative to the fit of a single factor model (all items across constructs load onto one factor) and a correlated factors model (each of the four latent factors was allowed to correlate). All models were evaluated using a Weighted Least Squares Mean and Variance Adjusted (WLSMV) estimator and model fit was determined based on a combination of a Comparative Fit Index (CFI) ≥ 0.95, an Standardized Root Mean Square Residual (SRMR) ≤ 0.08 (Hu & Bentler, 1999), and a Root Mean Square Error of Approximation (RMSEA) ≤ 0.08 (MacCallum et al., 1996). The higher‐order model provided a good overall fit to the data (*χ* ^2^(163) = 235.76, *p* = 0.002, RMSEA = 0.04, CFI = 0.99, SRMR = 0.06) and fit equally well^3^ as the correlated factors model (Δ*χ* ^2^(2) = 4.11, *p* = 0.13). Standardized factor loadings may be seen in Table S1.

Our preregistered analysis plan for analyses subsequent to those described above and explained in detail in Christophe et al. (2021) are available via the Open Science Framework (https://osf.io/5vmqc). After deciding on the higher‐order factor model, factor scores, or approximate scores for each person on our higher‐order factor, culturally informed S&P were used as indicators in our subsequent regression models (Ng & Chan, 2020). Predictors in these models included discrimination, culturally informed S&P, ERI, and their interactions (see Table 1). All predictors were mean‐centered before creating product terms.

**Table 1:** Standardized regression coefficients (*N *= 340)

	Outcome (estimator)								
	Anxiety (ML)			Binge drinking (WLSMV)			Quality sleep (WLSMV)		
Variable	*b* (SE)	*p*	95% CI	*b* (SE)	*p*	95% CI	*b* (SE)	*p*	95% CI
Discrimination	0.28 (0.05)	<0.001	0.17–0.38	0.03 (0.11)	0.80	−0.19 to 0.24	−0.11 (0.06)	0.05	−0.22 to 0.00
Culturally informed S&P	−0.21 (0.06)	<0.001	−0.32 to −0.10	−0.13 (0.09)	0.14	−0.30 to 0.04	0.35 (0.05)	<0.001	0.25 to 0.45
ERI	−0.05 (0.06)	0.40	−0.16 to 0.06	0.12 (0.10)	0.24	−0.08 to 0.31	−0.12 (0.05)	0.024	−0.26 to 0.02
Disc × Culturally informed S&P	−0.09 (0.06)	0.09	−0.20 to 0.01	−0.02 (0.13)	0.85	−0.27 to 0.22	0.11 (0.06)	0.06	−0.002 to 0.22
Disc × ERI	−0.02 (0.06)	0.77	−0.13 to 0.09	−0.02 (0.14)	0.87	−0.30 to 0.25	−0.14 (0.06)	0.02	−0.25 to 0.03
Culturally informed S&P × ERI	0.004 (0.06)	0.95	−0.15 to 0.12	−0.03 (0.09)	0.76	−0.21 to 0.15	−0.05 (0.06)	0.36	−0.17 to 0.06
Disc × Culturally informed S&P × ERI	−0.01 (0.06)	0.86	−0.13 to 0.11	0.02 (0.17)	0.89	−0.30 to 0.35	−0.06 (0.07)	0.40	−0.19 to 0.07
Model *R* ^2^	*R* ^2^ = 0.13, *p* < 0.001			*R* ^2^ = 0.03, *p* = 0.38			*R* ^2^ = 0.13, *p* < 0.001		

Abbreviations: ML, maximum likelihood; WLSMV, Weighted Least Squares Mean and Variance Adjusted.

### 4.2 Anxiety

We treated our sum score of anxiety (*M* = 8.77, SD = 8.58, range *=* 0–36) as a continuous outcome and used a maximum likelihood estimator, and treated missing data using full information maximum likelihood (FIML). Skewness (1.09) and kurtosis (0.50) were acceptable. Overall, our predictors accounted for 13% of the variation in anxiety symptoms (*p* < 0.001). Results from this regression model indicated that discrimination was associated with greater anxiety symptoms (*b* = 0.28, *p* < 0.001), while culturally informed S&P promoted fewer anxiety symptoms (*b* = −0.21, *p* < 0.001). Consistent with hypotheses, the association between discrimination and anxiety was not moderated by culturally informed S&P (*b* = −0.09, *p* = 0.09). There were no other significant interactions.

### 4.3 Binge drinking

While we intended to use a maximum likelihood estimator to handle our categorical binge drinking outcome, we observed a floor effect (79.7% of values indicated no binge drinking over the last 2 weeks) when examining the distribution. Based off recommendations by Muthén (2001), we therefore, used a WLSMV estimator to better handle this skewed (skewness = 2.57) distribution. Overall, our predictors accounted for a nonsignificant amount of variance in the underlying response variable of binge drinking (*R* ^2^ = 0.03, *p* = 0.38). Similarly, and contrary to hypotheses, none of our variables nor our interactions were associated with binge drinking.

### 4.4 Quality sleep

**Figure 1:**
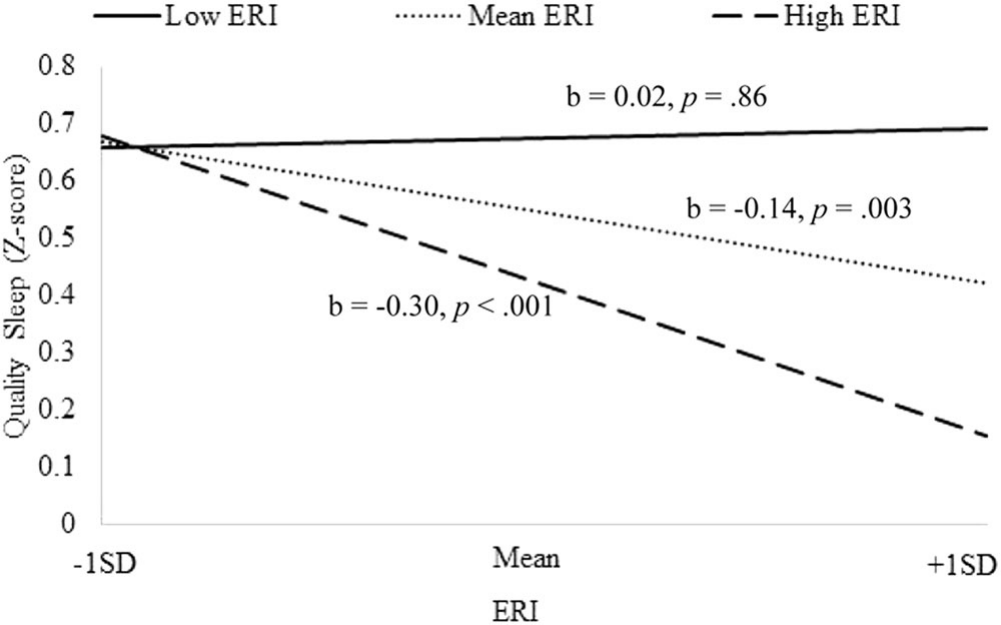
Simple slopes plot predicting quality sleep. *β*'s are probit coefficients. *b* = −0.30, *p* < 0.001

While we intended to use a distribution designed for count variables (e.g., Poisson, negative binomial, etc.) to examine quality sleep, our response options asking participants how many days in the last week they had gotten restful sleep ranged from 1–7 days. Without a true zero response option, we were unable to treat this variable as count and, thus, treated it as categorical. We observed a floor effect where a sizeable percentage of participants (31.9%) indicated that they had only had one quality night of sleep in the past 7 days. We, therefore, used a WLSMV estimator to better account for this floor effect (Muthén, 2001).^4^ Overall, our predictors explained 13% of the variance in the underlying response variable for quality sleep (*p* < 0.001). In terms of main effects, culturally informed S&P promoted more frequent quality sleep (*b* = 0.35, *p* < 0.001). Discrimination (*b* = −0.11, *p* = 0.05) and ERI (*b* = −0.12, *p* = 0.024) were associated with less quality sleep. Contrary to hypotheses, there was not a significant interaction between discrimination culturally informed S&P (*b* = 0.11, *p* = 0.06). There was, however, a significant interaction between discrimination and ERI (*b* = −0.14, *p* = 0.02). Probing this interaction (see Figure 1) revealed that discrimination does not interfere with quality sleep for those low in ERI (*b* = 0.02, *p* = 0.86), but was associated with less quality sleep for those at mean (*b* = −0.14, *p* = 0.003), and high levels of ERI (*b* = −0.30, *p* < 0.001). There were no other significant two‐ or three‐way interactions.

### 4.5 Sensitivity analysis

To examine the consistency of our findings, we conducted several sensitivity analyses. First, we reran our three models including age, gender, subjective social status, and nativity status as covariates. Findings for anxiety and binge drinking remained the same and none of our four predictors were significantly associated with outcomes (see Table S2). However, we observed small differences when examining predictors of quality sleep. In this model, higher subjective social status (*b* = 0.15, *p* = 0.006) was associated with better quality sleep, while greater age (*b* = −0.16, *p* = 0.008) and female gender (*b* = −0.11, *p* = 0.042) were associated with less frequent quality sleep. ERI was still associated with less quality sleep (*b* = −0.11, *p* = 0.045), and ERI still interacted with discrimination to predict quality sleep (*b* = −0.13, *p* = 0.023), but the main effect of discrimination was no longer significant (*b* = −0.08, *p* = 0.112). Additionally, due to our findings regarding the null impact of discrimination on binge drinking, which run counter to the positive associations found in previous research (e.g., Metzger et al., 2018), we conducted a second sensitivity analysis substituting our measure of binge drinking with a one‐item measure asking participants “on the occasions that you drink alcoholic beverages, how often do you drink enough to feel pretty drunk?” Participant responded to this drinking to intoxication frequency item on a 1 (*none*) to 5 (*nearly all*). This model was run using a WLSMV estimator and included all four previously mentioned demographic covariates. Culturally informed S&P promoted less frequent drinking until intoxication (*b* = −0.18, *p* = 0.016), while ERI (*b* = 0.17, *p* = 0.021) and age (*b* = 0.18, *p* = 0.002) were associated with greater intoxication frequency. No other predictors nor interactions between predictors were associated with frequency of intoxication (see Table S3).

## 5 DISCUSSION

Building on Neblett et al.'s (2012) theoretical model asserting that multiple cultural and normative factors work in tandem to provide minoritized youth with resilience in the face of discrimination, the present study was the first to examine whether marginalized individual's use of culturally informed S&P coping is protective in the face of discrimination and/or promotive of anxiety and two important health behaviors, binge drinking, and quality sleep. Although we found no evidence of protective effects, there is emerging evidence of culturally informed S&P promoting better health outcomes when taking into account ERI and discriminatory experiences, as culturally informed S&P was associated with less anxiety and better self‐reported sleep as main effects. Broadly, our findings demonstrating empirical support for the existence of culturally informed S&P, an underlying factor that represents the commonalities among shift, persist, civic engagement, and spiritually based coping, support Neblett et al.'s (2012) theory regarding the ways in which promotive cultural and normative factors relate to one another. However, our findings also diverge from theory, as we do not find that culturally informed S&P protects against the harmful impact of discrimination on anxiety, binge drinking, and quality sleep. These findings also diverge from previous work done with this sample finding that culturally informed S&P protected against the impacts of discrimination on depressive symptoms (Christophe et al., 2021). Neblett et al.'s (2012) theory does not outline the specific outcomes and conditions when these cultural and normative factors are protective; it more generally asserts that these factors promote positive “adjustment” and reduces discrimination's impact on “adjustment.” Our findings, in combination with those from Christophe et al. (2021), thereby advance this theory by adding specificity and highlighting specific outcomes where these factors may and may not demonstrate promotive and protective effects.

Further, in sensitivity analyses, culturally informed S&P was also associated with less drinking until the point of intoxication. These main effects lend credence to the theoretical assertions that culturally embedded coping processes whereby youth engage in coping that is both consonant with their worldview and cultural identity and characterized by the ability to cognitively disengage from stressors and turn towards meaning and purpose foster well‐being and less risk minoritized youth (e.g., Heppner et al., 2014). Further, these processes are promotive across level of risk, in other words, these effects are not conditional on how much discrimination one faces. This suggests that unlike the traditional S&P literature whereby the protective benefits were only associated with positive health outcomes for those at the highest level of risk (e.g., lowest SES; Chen et al., 2015) that for minoritized youth these effects are protective all levels of risk likely because as minoritized youth their level of risk is already greater than higher‐SES nonminoritized youth. Future work should continue to test how S&P coping functions as a promotive and protective effect for both mental health and health outcomes more specifically for minoritized youth.

Although we found that culturally informed S&P coping was associated with less anxiety as a main effect, we did not find evidence that culturally informed S&P was protective or reduced the impact of discrimination on anxiety symptoms. These findings differ from what has been observed with depressive symptoms. In this same sample, Christophe et al. (2021) found that discrimination was no longer associated with depressive symptoms when participants used high levels of culturally informed S&P coping and was generally promotive of less depression as a main effect. While both sets of findings suggest that culturally informed S&P coping is adaptive and associated with fewer symptoms, this manner of coping may reduce the negative impact of depression because the acts of shifting, persisting, being civically engaged, and engaging in spiritually based coping may connect someone facing discrimination with the affirming and mood‐boosting properties of one's cultural beliefs, values and traditions. This process has been coined by Mosley et al. (2020) as radical hope, or the “steadfast belief in the collective cap city contained within communities of color to heal and transform oppressive forced into a better future” (p. 3). By understanding their history of oppression and resistance while envisioning future possibilities through civic engagement and at the same time doubling down on the pride of those ancestral pride and faith through spiritual/religious coping (Mosley et al., 2020)—synergistically driving meaning and purpose—these coping strategies may be psychologically buffering youth against the typical impact discrimination would have on one's affect.

Anxiety, however, differs from depression, and effective coping and the maintenance of radical hope does not alter the fact that marginalized individuals are likely to continue to experience this same discrimination repeatedly throughout their lifetimes. We would, therefore, not expect the impact of discrimination on anxiety to be lessened by effective coping because each exposure to discrimination continuously fuels anxieties about future exposures to discrimination. Race‐based rejection sensitivity theory supports this finding and explains the process by which discrimination may result in increased hypervigilance, expectation of discrimination, and strong subsequent reactions to discrimination (Mendoza‐Denton et al., 2002). The measure of anxiety used in the current study from the Depression Anxiety Stress Scale‐21 (DASS‐21) (Lovibond & Lovibond, 1995) places special emphasis on physiological markers of anxiety which may be characteristic of those who, regardless of how well they may recover over time through effective coping, may be physiologically aroused in anticipation of future instances of discrimination. Race‐based rejection sensitivity inherently involves strong stress reactions to discrimination or race‐based rejection (Garthe et al., 2020), which are likely many the same physiologically based anxiety symptoms we are assessing with the DASS‐21. Future studies may benefit from explicitly examining how factors such as race‐based rejection sensitivity and experiences of discrimination covary, snowball, and impact anxiety symptomatology throughout development. When examining the effects of discrimination, parsing out differences between its impact of race‐based rejection sensitivity versus other dimensions of clinical anxiety (e.g., cognitive vs. physiological dimensions) may be important, as coping factors and cultural assets may not be equally promotive or protective across construct and dimension. This differentiation of cognitive versus physiological aspects of anxiety may be useful in teasing apart the contexts in which Neblett et al.'s (2012) theoretical model holds, and these factors do lead to fewer symptoms of anxiety and disrupt discrimination's harmful effects on anxiety.

Interestingly, we did find evidence for an intensifying effect where discrimination decreased the frequency of quality sleep for those with average to strong ERIs. This finding runs counter to Yip et al.'s (2020) finding that high ERI commitment (knowing what race/ethnicity means to you) was associated with better sleep among adolescents who experienced discrimination. However, a few notable differences between these two studies may explain these disparate findings. First, the sample in Yip et al.'s (2020) study is younger (*M*_age_ = 14.29) compared to our study featuring college students (*M*_age_ = 18.79). The impact of factors such as discrimination and identity may differentially affect youth at different stages of development, and notable biologically and socially driven differences in sleep patterns between adolescents and emerging adults have been documented (Wolfson, 2010).

Second, our measure of ERI, which was derived from an adapted version of the MIBI, centers on the centrality and private regard rather than the ERI commitment subscale of Phinney's Multigroup Ethnic Identity Measure (1992) that Yip et al. (2020) used. In their earlier meta‐analysis of the protective effects of different ERI dimensions, Yip et al. (2019) observed that, unlike private regard and centrality, commitment broadly buffered the discrimination‐general adjustment association. Third, our measurement of sleep is limited in that it consists of one self‐reported item compared to the multidimensional and objective measurement utilized by Yip et al. (2020). In our study, we may have observed what has been called the “double‐edged sword” of ERI (Yip, 2018), where discrimination is more harmful for high ERI youth because the identity being discriminated against is a crucial part of these youth's self‐concept. Similar to what was found with anxiety, while culturally informed S&P may promote more frequent quality sleep as a main effect, other protective factors may be needed to reduce the negative effect of discrimination on sleep, especially among youth with strong ERIs whose sleep is particularly affected by this discrimination. Alternatively, sleep has been posited as a coping mechanism in and of itself or one that might encourage positive coping when individuals encounter discrimination. For example, among a sample of 256 minoritized adolescents, longer and better sleep on the previous night before a discriminatory encounter was linked to adolescents’ use of greater active coping (Wang & Yip, 2020). Future studies should attend to both the potential buffering effects of sleep and the disruption of sleep due to discrimination experiences.

Related to binge drinking, we were surprised by the null effect between discrimination and binge drinking as there is emerging evidence that discrimination is linked to greater drinking in college samples (e.g., Metzger et al., 2018; Su et al., 2020). We believe this may be related to timeframe for which the data were collected. We assessed binge drinking in the last 2 weeks while both the Metzger et al. (2018) and Su et al. (2020) studies assessed drinking in the past year. Further, another study that did not find a discrimination‐binge drinking link in Black college students also used a shorter timeframe in the measure (past month use, Desalu, Kim, et al., 2019). It may be that due to the infrequency of binge drinking (as seen in our data) that this effect is best captured when the timeframe allows for more variability in binge drinking, particularly for minorized youth. There was a slight indication of a promotive effect of S&P coping when looking at intoxication in our sensitivity analyses, but largely, more work needs to test how S&P coping may influence drinking outcomes with deeper measures of alcohol use, across wider time frames, and potentially taking into account consequences of alcohol use as well (Desalu, Goodhines, et al., 2019).

### 5.1 Limitations

While this study contributes to a growing literature examining the nexus between culturally informed coping, ERI, and discrimination, this study is not without its limitations. The cross‐sectional design utilized in the current study limits our ability to make claims regarding directionality and causality. In reality, it is likely that culturally informed S&P and ERI predict outcomes in the way we have modeled, but also that one's symptomatology and health behaviors then have an impact of one's subsequent coping ability. Longitudinal work is needed to examine the transactional relations between these factors across development. Furthermore, while we relied on single‐item measures of binge drinking and sleep that have been used extensively in nationally representative surveys and have high face validity, it is possible that multiple‐item self‐reported measures with strong, established psychometric properties (e.g., the Pittsburgh Sleep Quality Index; Buysse et al., 1989) may provide broader representations of our constructs of interest with greater construct and external validity. Employing these types of measures may give researchers a greater ability to identify significant and meaningful effects if they exist in the population.

## 6 CONCLUSIONS

In conclusion, this study advances our understanding of S&P coping and its related culturally informed coping strategies by examining when and for what mental health and health behavior outcomes culturally informed S&P is promotive and protective. Furthermore, our findings both support Neblett et al.'s (2012) theory speaking to the importance of simultaneously examining the impact of multiple resilience factors and diverge from theory by illustrating several outcomes for which the joint impact of cultural and normative resilience factors does not protect against discrimination. While culturally informed S&P was not protective in the face of discrimination for anxiety, binge drinking, and sleep, it did generally promote less anxiety and more frequent quality sleep. Furthermore, we found additional evidence that discrimination is a particularly strong disruptor of sleep for those with moderate to strong ERIs. By coming to a more nuanced understanding of when S&P‐style coping is effective, for whom it is effective, and for what outcomes it is promotive and/or protective, we may better support youth in their coping efforts and support resilience in minoritized youth across outcomes and contexts. Further, understanding the contexts in which coping and ERI do and do not interface to promote positive functioning and protection in the face of discrimination may be used to further refine theory and, ultimately, intervene and create contexts that support minoritized youth's continued wellbeing and resilience.

## CONFLICT OF INTERESTS

The authors declare that there are no conflict of interests.

### PEER REVIEW

The peer review history for this article is available at https://publons.com/publon/10.1002/jcop.22799

## Supporting information

Supporting information.

## Data Availability

Research data are not shared.

## Footnotes

1 For brevity, we subsequently refer to racially and ethnically minoritized individuals solely as minoritized individuals/youth/and so on.

2 A sixth shift item, “When something doesn't turn out the way I want, I think about what good things could come from the situation” was not included in the study due to a survey coding error. Given the high degree of wording overlap with the shift item presented as a sample item, the authors judged that the remaining five items adequately assessed the shift construct and continued with analyses.

3 The higher‐order factor model was nested within the correlated factors model; therefore, the higher‐order model can fit significantly worse than the correlated factor model, but cannot fit significantly better (Brown, 2015).

4 Interpretation of path coefficients remained the same when comparing results using WLSMV to results using maximum likelihood. However, because the WLSMV is recommended over maximum likelihood when there are floor effects among categorical outcome variables (Muthén, 2001), we report regression coefficients using the recommended WLSMV estimator.
