# Supplementary material for: Examining the promotive versus the protective impact of culturally informed shift‐&‐persist coping in the context of discrimination, anxiety, and health behaviors
Source: J Community Psychol. 2022 Jan 20;50(7):2829–44. doi: 10.1002/jcop.22799 (PMC9296692; doi:10.1002/jcop.22799)
Supplement: Supplementary file 1 — Supporting information. [file JCOP-50-2829-s001.docx]

**Supplemental Material**

| **Table S1**  *Model Fit Indices and Standardized Factor Loadings.* | | | | |
| --- | --- | --- | --- | --- |
| Fit Index | Higher-Order Factor Model | | | |
| χ^2^ (*df*) | 235.76 (163) | | | |
| *p*-value | .0002 | | | |
| RMSEA [90% CI] | .035 [.025 - .045] | | | |
| CFI | .99 | | | |
| TLI | .99 | | | |
| SRMR | .06 | | | |
| 2^nd^ order factor loading | Persist | Shift | Civ | Spir |
| Culturally-informed S&P | .78 | .82 | .17 | .38 |
|  |  |  |  |  |
| 1^st^ order factor loading |  |  |  |  |
| Persist 1 | .816 |  |  |  |
| Persist 2 | .800 |  |  |  |
| Persist 3 | .307 |  |  |  |
| Persist 4 | .876 |  |  |  |
| Persist 5 | .599 |  |  |  |
| Persist 6 | .420 |  |  |  |
| Persist 7 | .696 |  |  |  |
| Shift 1 |  | .794 |  |  |
| Shift 2 |  | .827 |  |  |
| Shift 4 |  | .729 |  |  |
| Shift 5 |  | .913 |  |  |
| Shift 6 |  | .700 |  |  |
| Civ 1 |  |  | .665 |  |
| Civ 2 |  |  | .858 |  |
| Civ 3 |  |  | .594 |  |
| Civ 4 |  |  | .947 |  |
| Spir 1 |  |  |  | .954 |
| Spir 2 |  |  |  | .933 |
| Spir 3 |  |  |  | .707 |
| Spir 4 |  |  |  | .967 |

*Note.* All models were created using the WLSMV (Weighted Least Square Mean and Variance Adjusted) Estimator. All standardized factor loadings are significant at p < .05. Civ = Critical Civic Engagement. Spir = Spiritually-based coping. A Shift item, item 10 in Lam et al.’s 13-item shift-&-persist scale (2018) was missing due to a survey programming error.

| **Table S2**  *Standardized Regression Coefficients including demographic covariates (N=340).* | | | | | | | | | | | | |
| --- | --- | --- | --- | --- | --- | --- | --- | --- | --- | --- | --- | --- |
|  |  |  | | Outcome (Estimator) | | | | | | | | |
|  | Anxiety (ML) | | | |  | Binge Drinking (WLSMV) | | |  | Quality Sleep (WLSMV) | | |
| Variable | b (*S.E.*) | *p* | 95% *CI* | |  | b (*S.E.*) | *p* | 95% *CI* |  | b (*S.E.*) | *p* | 95% *CI* |
| Discrimination | 0.26 (0.05) | <.001 | 0.16-0.37 | |  | 0.02 (0.11) | .829 | -0.20-0.25 |  | -0.08 (0.05)* | .112 | -0.19-0.02 |
| Culturally-informed S&P | -0.20 (0.06) | <.001 | -0.31- -0.09 | |  | -0.16 (0.09) | .089 | -0.34-0.03 |  | 0.29 (0.05) | <.001 | 0.19-0.40 |
| ERI | -0.05 (0.06) | .410 | -0.16-0.07 | |  | 0.16 (0.10) | .112 | -0.04-0.36 |  | -0.11 (0.06) | .045 | -0.22- -0.002 |
| Disc * Culturally-informed S&P | -0.10 (0.06) | .088 | -0.20-0.01 | |  | -0.01 (0.13) | .954 | -0.26-.25 |  | 0.08 (0.06) | .134 | -0.03 – 0.19 |
| Disc * ERI | -0.03 (0.06) | .660 | -0.13-0.09 | |  | -0.02 (0.14) | .886 | -0.30-0.26 |  | -0.13 (0.06) | .023 | -0.24- -0.02 |
| Culturally-informed S&P * ERI | 0.01 (0.06) | .917 | -0.11-0.12 | |  | -0.04 (0.09) | .689 | -0.22-0.15 |  | -0.08 (0.06) | .188 | -0.20-0.04 |
| Disc * Culturally-informed S&P * ERI | -0.02 (0.06) | .761 | -0.14-0.10 | |  | 0.03 (0.17) | .844 | -0.31-0.38 |  | -0.08 (0.06) | .217 | -0.20-0.05 |
| Age | 0.09 (0.05) | .087 | -0.01-0.19 | |  | 0.11 (0.08) | .179 | -0.05-0.28 |  | -0.16 (0.06) | .008 | -0.28--0.04 |
| Gender | 0.02 (0.05) | .667 | -0.08-0.13 | |  | -0.07 (0.08) | .392 | -0.22-0.08 |  | -0.11 (0.05) | .042 | -0.21--0.004 |
| Subjective Social Status | -0.01 (0.05) | .803 | -0.12-0.09 | |  | 0.04 (0.09) | .643 | -0.14-0.22 |  | 0.15 (0.05) | .006 | 0.04-0.26 |
| U.S. Born | 0.05 (0.05) | .354 | -0.05-0.15 | |  | 0.14 (0.10) | .168 | -0.06-0.33 |  | 0.01 (0.06) | .909 | -0.10-0.12 |
| Model *R^2^* | *R^2^* = .14, *p* < .001 | | | |  | *R^2^* = .07, *p* = .179 | | |  | *R^2^* = .18, *p* < .001 | | |

*Note.* * denotes an effect that changed in significance following the introduction of covariates.

**Table S3**

*Standardized Regression Coefficients Predicting Intoxication Frequency.*

|  | *Intoxication Frequency*(WLSMV) | | |
| --- | --- | --- | --- |
| Variable | b (*S.E.*) | *p* | 95% *CI* |
| Discrimination | -0.01 (0.07) | .881 | -0.15-0.13 |
| Culturally-informed S&P | -0.18 (0.07) | .016 | -0.32--0.03 |
| ERI | 0.17 (0.08) | .021 | -0.03-0.32 |
| Disc * Culturally-informed S&P | -0.01 (0.07) | .885 | -0.15-0.13 |
| Disc * ERI | 0.06 (0.09) | .515 | -0.11-0.23 |
| Culturally-informed S&P * ERI | -0.01 (0.07) | .864 | -0.14-0.12 |
| Disc * Culturally-informed S&P * ERI | 0.11 (0.08) | .179 | -0.05-0.27 |
| Age | 0.18 (0.06) | .002 | 0.07-0.30 |
| Gender | 0.01 (0.06) | .865 | -0.11-0.14 |
| Subjective Social Status | 0.08 (0.07) | .202 | -0.04-0.21 |
| U.S. Born | 0.13 (0.07) | .069 | -0.01-0.27 |
| Model *R^2^* | *R^2^*= 0.08, *p* = .023 | | |

*Note.* WLSMV = weighted least squares mean and variance adjusted estimator. S&P = Shift-&-Persist. Disc = Discrimination. ERI = Ethnic-racial Identity.
